# Investigating relationships among coping, personal growth, and life satisfaction among individuals with physical disabilities

**DOI:** 10.34172/hpp.2020.59

**Published:** 2020-11-07

**Authors:** Junhyoung Kim, Areum Han, Jennifer Ann Piatt, Jaehyun Kim

**Affiliations:** ^1^School of Public Health, Indiana University Bloomington, IN, USA; ^2^Center for Curriculum and Institute of Studies, Korea University, Seoul, South Korea; ^3^Department of Recreation, Therapeutic Recreation, and Tourism, State University of New York, College at Brockport, NY, USA

**Keywords:** Psychological, Coping behaviors, Personal satisfaction, Growth, Disabled persons

## Abstract

**Background:** It is well-documented that active coping strategies can lead to better positive adjustment and psycho-social outcomes among individuals with disabilities and illnesses. However, little information exists related to exploring how coping is related to health benefits such as personal growth and life satisfaction in an international context. Thus, this study examined how the use of coping strategies is associated with personal growth and life satisfaction among individuals with physical disabilities in non-Western settings.

**Methods:** In this cross-sectional study, we employed a nonprobability methodology, purposivesampling, to recruit 351 participants who adults over the age of 18 living with a physicaldisability and currently enrolled in the Korean Disability Association. A hierarchical linearregression analysis was conducted to determine which types of coping strategies predictedpersonal growth and life satisfaction, while controlling for the effects of the demographicvariables (i.e., gender and age).

**Results:** With regard to personal growth, problem-solving coping strategy (β = 0.663, P < 0.001,95% CI [0.51, 0.70]) was the strongest predictor, followed by avoidance coping strategy(β = -0.263, P < 0.001, 95% CI [-0.37, -0.20]). As for life satisfaction, problem-solving copingstrategy (β = 0.268, P < 0.001, 95% CI [0.18, 0.70]) was the strongest predictor, followed bysocial support seeking coping strategy (β = 0.264, P < 0.001, 95% CI [-0.19, 0.10]).

**Conclusion:** Our study suggests that problem-focused and social support coping strategies playimportant roles in improving the personal growth and life satisfaction among Korean individualswith physical disabilities. This study provides implications for health professionals seeking waysto facilitate the personal growth and enhance the life satisfaction of individuals with physicaldisabilities.

## Introduction

The Ministry of Health and Welfare^1^ reported that over 1.3 million people living in South Korea were diagnosed with physical disabilities, representing approximately 50% of the disabled community. This population is expected to continue to increase annually.^2^ Prior studies have demonstrated that individuals with physical disabilities often experience a variety of physical, social, and psychological challenges associated with their limited physical functioning, resulting in diminished life satisfaction.^3,4^ In addition, such challenges often generate high levels of stress and psychological distress. Thus, healthcare professionals have suggested that developing effective coping strategies to confront and manage their challenges can help individuals with physical disabilities improve their mental health and quality of life.^5-7^

A growing body of literature focuses on the types of coping strategies (e.g., active coping and passive coping) that are associated with health outcomes among individuals with physical disabilities.^8-11^ Previous findings have provided evidence that active coping strategies can lead to better positive adjustment and psycho-social outcomes among individuals with disabilities and illnesses, whereas avoidance or passive forms of coping predict increased psychological distress and diminished mental health.^12-14^ For example, Livneh and Martz^8^ showed that individuals with spinal cord injuries who used active engagement in coping strategies (e.g., positive reframing, acceptance seeking social support) experienced better psycho-social adjustment and quality of life outcomes than those who did not.

In physical rehabilitative settings, health care providers help patients achieve personal growth and life satisfaction through various therapeutic programs. Personal growth involves self-understanding and the ability to manage stressful situations.^15,16^ In addition, the life satisfaction of individuals with physical disabilities has been found to be strongly related to such health benefits as improved mental health and longevity in various clinical settings.^17,18^

In spite of the global scope of issues connected with physical disability, the majority of previous studies have focused on the relationship between coping and health benefits among Western individuals with physical disabilities such as spinal cord injury, arthritis, and multiple sclerosis.^8-10^ In an international context, little information exists related to exploring how coping is related to health benefits such as personal growth and life satisfaction. For international and particularly non-Western audiences, it is important to explore the relationship among coping, personal growth, and life satisfaction within the contexts of their cultures. Therefore, the purpose of this study was to examine the extent to which the use of coping strategies contributed to personal growth and life satisfaction among Korean individuals with physical disabilities.

### Coping theory

Folkman et al^19^ defined coping as a “person’s cognitive and behavioral efforts to manage (reduce, minimize, master, or tolerate) the internal and external demands of the person-environment transaction that is appraised as taxing or exceeding the person’s resources” (p. 572). Lazarus and Folkman^20^ identified two ways of coping with life stressors as problem-focused and emotion-focused. They stated that problem-focused coping involves directly and actively dealing with life stressors in a practical manner, whereas emotion-focused coping involves efforts to regulate psychological distress and emotions rather than directly addressing the stressful situations. To this bi-dimensional construct of strategies for coping with life stressors, Folkman and Lazarus^21^ added a third category, seeking social support.

Amirkhan^22^ redefined coping strategies as problem-solving/active coping, seeking social support, and avoidance/withdrawal. In this construct, problem-solving is considered to be active coping as it involves active management of stressors and their effects. He defined seeking social support as a process of actively seeking help, comfort, and advice to cope with stressful situations. Avoidance involves physical and/or psychological withdrawal from stressful situations. Kara and Açıkel^23^ found that individuals with physical disabilities most often used problem-solving coping strategies, followed by avoidance and seeking social support. Moreover, they found that physically disabled individuals with financial challenges were more likely to use avoidance, whereas those who had caregivers and experienced high levels of social support were more likely to seek social support.

To conceptualize coping, Amirkhan’s^22^ methods to assess three coping strategies (i.e., problem-solving/active coping, seeking social support, and avoidance/withdrawal) were adopted in this study. In a variety of clinical contexts, Amirkhan’s coping approach has been applied across various populations such as veterans with limb amputations and women with posttraumatic stress disorder.^24,25^

### Coping, personal growth, and life satisfaction

Prior studies have provided evidence that coping strategies contribute to personal growth among individuals who have experienced traumatic life events.^26-28^ In their meta-analysis of 103 studies of factors contributing to personal growth, Prati and Pietrantoni,^29^ found that specific coping strategies (e.g., seeking social support, religious coping, acceptance, positive reappraisal) were positively associated with personal growth. In addition, Arikan and Karanci^26^ explored the relationship between coping strategies and personal growth among college students who had experienced traumatic events. They indicated that seeking social support and strategies for overcoming fatalistic attitudes were predictive of personal growth.

The importance of active and practical involvement in dealing with stressors along with social support is a theme throughout the literature. Litman and Lunsford^30^ examined the contributions of three types of coping strategies (i.e., self-sufficient approach-oriented coping, socially-supported approach-oriented coping, avoidant-oriented coping) to personal growth or diminishment. They found that an increase in the use of self-sufficient approach-oriented strategies (i.e., acceptance, planning) led to an increase in personal growth, while an increase in the use of socially-supported approach-oriented coping (e.g., emotional support) led to a decrease in stressful situations. Mikula et al^6^ found psychological coping strategies such as consciously stopping unpleasant emotions and thoughts were major contributors to the mental quality of life of patients with multiple sclerosis. Pereira et al^11^ demonstrated that active coping, religious faith, acceptance, and humor were significantly associated with improved life satisfaction of individuals with lower limb amputations.

Previous findings have suggested that developing various types of coping strategies that combine self- and social-reliance is positively associated with life satisfaction and quality of life.^31-33^ For example, Luque et al^34^ found that the use of problem-solving, social support, and self-expression as coping strategies resulted in improved life satisfaction of patients in their study. Similarly, Persson and Rydén^35^ found that engaging in active behaviors to deal with problems, minimizing their negative effects, and trusting in self and society were effective coping strategies among individual with physical disabilities.

Overall, these studies provide strong evidence that active coping plays an important role in improving mental health and psychological well-being among individuals with physical disabilities. The present study extends this body of research to the Korean context.

## Materials and Methods

### Population

In this cross-sectional study, we employed a nonprobability methodology and purposive sampling to recruit participants. They were adults over the age of 18 who were diagnosed with a physical disability and currently enrolled in the Korean Disability Association. Data were collected via a self-administered paper-pencil survey on site with the cooperation of the Korean Disability Association in South Korea during the period January 2018 to March 2018. Institutional Review Board approval was granted by the involved university and agency prior to any data collection. Participation in the study was voluntary with the option to withdraw from the survey at any time.

The sample consisted of 351 participants: 238 males (67.8%) and 113 females (32.2%). The sample ranged in age from 18 to 83 years, with a mean age of 54.4 years (SD = 11.53). The majority were married (64.4%), followed by single (17.9%), divorced (12.3%), and widowed (5.4%). With regard to levels of education, 35% had completed high school, 26.5% had graduated from college, and 3.1% held master’s or professional degrees. (see Table 1).

### Measurements

The survey used in this study comprised selected items from three previous instruments to measure personal growth, life satisfaction, and coping strategies. All items consisted of statements to be rated on a 7-point Likert type scale (1 = “very strongly disagree” to 7 = “very strongly agree”).

#### Personal growth

Seven items from the Measures of Psychological Well-Being instrument,^36^ previously used in rehabilitation sciences,^37^ were used to measure the construct of personal growth, with statements such as “I think it is important to have new experiences that challenge how I think about myself and the world” and “I have the sense that I have developed a lot as a person over time”. Higher scores indicated higher levels of personal growth among persons with physical disabilities. The Cronbach’s alpha for this measurement was 0.719.

*Life satisfaction*

Five items from a modified version of Diener et al’s^38^ Satisfaction with Life Scale (SWLS) were used to measure overall life satisfaction (e.g., “The conditions of my life are excellent”) Higher scores indicated greater life satisfaction. The Cronbach’s alpha for this scale was 0.854.

*Coping strategies*

Thirty-three items from Amirkhan’s^22^ Coping Strategy Indicator (CSI), consisting of three subcomponents: problem-solving, seeking social support, and avoidance, were used in this study. An example of problem-solving items is the statement, “When I am in trouble, I try different ways to solve the problem until I find one that works.” Seeking social support items included statements such as “When I am in trouble, I talk to people about the situation because talking about it makes you feel better.” Avoidance items included such statements as “When I am in trouble, I avoid being with people in general.” A Kaiser-Meyer-Olkin (KMO) measure of sampling adequacy showed a value of 0.910 (Bartlett’s sphericity = 4797.325, *P* <0.001), which indicated that the sample size was sufficient for the current study.^39^

As a result of the exploratory factor analysis (EFA), three subcomponents (i.e., problem-solving, seeking social support, avoidance) were extracted as they had eigenvalues greater than 1.00 and accounted for 50% of the extracted variance. However, two items (18 and 22), which had a communality criterion of 0.4 or less, were excluded at the outset 40, and three items (4, 6, and 10) that should belong to the avoidance sub-component of coping strategies 19 were excluded because these items actually belonged to the problem-solving sub-component of coping strategies.

An acceptable cut-off value for factor loading is 0.404, and the loading values for the factors of coping strategies ranged from 0.455 to 0.809 in this study. Therefore, a total of 28 items were used in the analysis. The Cronbach’s alpha for the problem-solving component was 0.922, for the seeking social support component was 0.868, and for the avoidance component was 0.630. Higher scores for each item indicated better coping strategies.

#### Data analysis

The Statistical Package for the Social Sciences (PAWS SPSS 18.0) was used for the data analysis. Descriptive statistics (e.g., means, SD) were generated to identify the demographic characteristics of the sample and a central tendency and dispersion of the study variables. Before the EFA, KMO and Bartlett’s sphericity tests were used to measure the sample’s adequacy. An exploratory factor analysis with principal component methods of factor extraction and a Varimax rotation and Cronbach’s alpha value of each construct were calculated in order to determine the validity and internal consistency of the measured variables. Pearson’s correlation coefficients were used to examine the relationships among coping strategies, life satisfaction, personal growth, and the demographic factors.^41^ The current study employed the tolerances and variance inflation factors (VIF) to detect multicollinearity. As a result, we found no multicollinearity issues among the independent variables (i.e., tolerance scores > 0.10; VIF < 10).^42^

Finally, a hierarchical linear regression analysis was used to determine which types of coping strategies predicted personal growth and life satisfaction. Specifically, based on a previous disability study, gender and age were noted as important demographic variables,^43^ so this study controlled for the effects of gender and age, which were therefore entered as the first step, and coping strategy types were entered as the second step, in the each of the two analyses. This two-block hierarchical regression analysis allowed us to examine the relative contributions of the study variables to the dependent variable, while controlling for the effects of the demographic variables.

## Results

As shown in Table 2, the mean scores were 4.84 (SD = 0.99) for problem-solving, 4.33 (SD = 0.90) for seeking social support, and 3.58 (SD = 0.89) for avoidance. The mean personal growth was 4.81 (SD = 0.95), indicating that participants generally indicated relatively high personal growth. The mean score for life satisfactionwas 3.88 (SD = 1.19).

Pearson’s correlation coefficients (Table 3) indicated that life satisfaction was positively related to problem-solving (*r* = 0.436, *P* < 0.01), seeking social support (*r* = 0.428, *P* < 0.01), and personal growth (*r* = 0.324, *P* < 0.01). Personal growth was positively associated with problem-solving (*r* = 0.614, *P* < 0.01) and seeking social support (*r* = 0.362, *P* < 0.01). However, personal growth was negatively associated with avoidance (*r* = -0.239, *P* < 0.01). Avoidance was negatively associated with age (*r* = –0.215, *P* < 0.01). Problem-solving was positively associated with age (*r* = 0.110, *P* < 0.05), indicating that the older adults with physical disabilities were more likely to use problem-solving strategies than younger counterparts.

After controlling for gender and age, hierarchical regression analyses were conducted to determine which types of coping strategies served as predictors of personal growth and life satisfaction. As for the personal growth (Table 4), results indicated that problem-solving (β = 0.633, *P* < 0.001) was the strongest predictor, followed by avoidance (β = -0.263, *P* < 0.001). Age (β = -0.102, *P* < 0.05) was a significant negative predictor of personal growth. In terms of life satisfaction (Table 5), results indicated that problem-solving (β = 0.268, *P* < 0.001) was the strongest predictor, followed by social support seeking (β = 0.264, *P* < 0.001). However, gender and age did not predict life satisfaction. Overall, the model explained 23.5% of the variance in life satisfaction and 44.7% of personal growth, while controlling for age and gender. The Durbin-Watson statistics were between 2.001 and 2.174, suggesting no autocorrelation in the sample.^44^

## Discussion

In this study, the relationships among types of coping strategies, personal growth, and life satisfaction for individuals with physical disabilities living in South Korea were explored. The results suggested that problem-solving served as the strongest predictor of personal growth. In addition, problem-solving and seeking social support were positively associated with life satisfaction. These results suggest that problem-focused and social support coping strategies play important roles in improving the personal growth and life satisfaction of individuals with physical disabilities living in this country.

Prior studies have stressed the importance of problem-solving as a means of promoting the health and well-being of individuals experiencing traumatic life events.,^28^ The results of this study expand the body of knowledge about problem-solving as an important factor in improving personal growth and life satisfaction among individuals with physical disabilities. Thus, it is suggested that helping individuals with physical disabilities develop problem-solving methods is an important rehabilitative strategy by which to promote their health and quality of life.

With reference to the international perspective mentioned above, Kara and Açıkel^23^ identified problem-solving as the most common coping strategy applied by Turkish individuals with physical disabilities, followed by avoidance and seeking social support. In this study, problem-solving was also the most common coping strategy though followed by seeking social support and avoidance. This difference may be explained by the effects of level of financial stability, age, and the caregiver’s presence on the coping strategies among individuals with physical disabilities.^23^

Some researchers have suggested that problem-solving can be maladaptive when the source of the stressors cannot be changed or eliminated.^45,46^ Considering the nature of the stressors that individuals with physical disabilities experience, they may encounter implacable challenges in changing or eliminating the problems associated with their limited physical functioning. However, the current study presents results showing that problem-solving is adaptive and beneficial for improving the personal growth and life satisfaction of individuals with physical disabilities, thus confirming the role of problem-solving in attenuating the stresses of their life events.

Consistent evidence has been presented that avoidance is linked to increased psychological and emotional distress, poor psychological adjustment, and diminished functioning skills among individuals with cancer and women with depression.^47,48^ The findings of this study are aligned with these studies and showed that for individuals with physical disabilities, avoidance is negatively associated with personal growth, which may be explained by underlying beliefs that they are unable to control their disabled conditions.

Researchers have stressed the importance of social support as a key component of lower depression and higher life satisfaction.^49,50^ The findings of this study are consistent these results and suggest that seeking social support is an effective means by which individuals with physical disabilities enhance life satisfaction. In a study of the impacts of coping strategies on mental and physical well-being, Englbrecht et al^51^ found that females with rheumatoid arthritis tended to use coping strategies (i.e., cognitive reframing, active problem-solving) more than males and that their use was positively associated with coping effectiveness, which led to a perception of increased general health. Similarly, Luque Salas et al^34^ found that females with ASD demonstrated higher expressions of emotions and levels of social support coping strategies than males with ASD. However, the current study showed that gender was not associated with coping strategies. With regard to age, older individuals with physical disabilities tended to use problem-solving, while younger individuals reported higher levels of seeking social support.

This study has several limitations. First, as Folkman et al^19^ indicated, coping responses are influenced by personal characteristics, social environments, and the nature and types of stressors. Future research is furthered to consider the nature and types of stressors or the social environments and examine their potential relationships with life satisfaction among persons with physical disabilities. In addition, the analysis in this study was based on Amirkhan’s framework of coping strategies,^22^ which does not take other coping strategies into account, such as religious coping, leisure/physical activity, and trust. Further studies, especially those employing qualitative research, are needed to explore various coping constructs or other items that the present study could not consider. Finally, this study did not explore the severity and types of physical disabilities among the participants that may be related to coping strategies and health benefits.

## Conclusion

This study provides implications for health professionals seeking ways to facilitate the personal growth and enhance the life satisfaction of individuals with physical disabilities. Health professionals need to design coping interventions for individuals with physical disabilities. By participating in coping skills training and interventions, individuals with physical disabilities can develop the ability to deal with various stressors in an active manner. In addition, health professionals need to implement programs and activities in which individuals with physical disabilities can receive positive social support from others. For example, the implementation of community-based programs can help individuals with physical disabilities connect with others and build a sense of friendship, decrease loneliness, and receive positive social support.

## Funding

No funding was received for this study.

## Competing interests

The authors declared no potential conflicts of interest with respect to the research, authorship, and/or publication of this article.

## Ethical approval

Not applicable.

## Authors’ contributions

The first author proposed a research idea and write on the sections of introduction and literature review. Then, first author had worked on analyzing the collected data with the second author, third author, and fourth author. AH as a corresponding author was responsible for writing material and methods and results sections. We as a research team worked together to write on the sections of discussion and implications and conclusions. Each author had contributed to the editing process throughout the whole manuscript. Finally, all authors have approved the manuscript for submission to the *Health Promotion Perspectives* .

**Table 1:** Participant characteristics

**Characteristics**	***n***	**%**
Age		
18-29	16	4.6
30-39	25	7.1
40-49	52	14.8
50-59	133	37.9
>60-69	103	29.3
70-83	22	6.3
Gender		
Male	238	67.8
Female	113	32.2
Marital status		
Married	226	64.4
Divorced	43	12.3
Single	63	17.9
Widowed	19	5.4
Education		
Middle school graduate	70	19.9
High school graduate	130	37.0
Some college	23	6.6
College graduate	93	26.5
Graduate college	11	3.1
Other	24	6.8

**Table 2:** Descriptive statistics of the measured variables

**Variables**	**Mean**	**Standard deviation**
Problem solving	4.84	0.99
Social support seeking	4.33	0.90
Avoidance	3.58	0.89
Personal growth	4.81	0.95
Life satisfaction	3.88	1.19

**Table 3:** Correlation matrix for all measurement variables

	**Gender**	**Age**	**PS**	**SSS**	**A**	**PG**	**LS**
Gender	-						
Age	0.104	-					
PS	0.040	0.110^*^	-				
SSS	-0.010	-0.028	0.620^**^	-			
A	0.023	-0.215^**^	0.007	0.101	-		
PG	-0.020	0.022	0.614^**^	0.362^**^	-0.239^**^	-	
LS	0.073	0.046	0.436^**^	0.428^**^	0.010	0.324^**^	-

Abbreviations: PS, problem solving; SSS, social support seeking; A, avoidance; PG, personal growth; LS, life satisfaction. Note. ** P < 0.01, * P < 0.05.

**Table 4:** Summary of Hierarchical Regression Analysis for Variables Predicting Personal Growth

	**Step 1**					**Step 2**				
	**B**	**β**	*t* **(Sig)**	**95% Confidence interval**		**B**	**β**	**t(Sig)**	**95%Confidence interval**	
				**Lower bonding**	**Upper bonding**				**Lower bonding**	**Upper bonding**
Block 1									
Gender	-.045	-.022	-.413 (*P* =.680)	-.261	.171	-.058	-.029	-.707 (*P* =.480)	-.220	.104
Age	.002	.024	.443 (P=.658)	-.007	.011	-.008	-.102	-2.441* (*P* <.05)	-.015	-.002
Block 2									
Problem Solving						.604	.663	12.219*** (*P* <.001)	.507	.702
Social Support Seeking						-.008	-.007	-.144 *P* =.885)	-.115	.099
Avoidance						-.282	-.263	-6.384*** (*P* <.001)	-.369	-.195
F	.166					55.850^***^				
R^2^	.001					.447				
R^2^_Change_	.005					.439				

Note: *P < .05, ***P <.001; Dummy variables: Gender (male=1, female=0)

**Table 5:** Summary of Hierarchical Regression Analysis for Variables Predicting Life Satisfaction

	**Step 1**					**Step 2**				
	**B**	***β***	***t*** **(sig)**	**95% Confidence interval**		**B**	***β***	**t(sig)**	**95% Confidence interval**	
				**Lower bonding**	**Upper bonding**				**Lower bonding**	**Upper bonding**
Block 1									
Gender	.176	.069	1.285 (*P* =.220)	-.093	.446	-.163	.064	1.346 (*P* =.179)	-.075	.104
Age	.004	.039	.724 (*P* =.470)	-.007	.015	.001	.014	.284 (*P* =.776)	-.009	-.002
Block 2									
Problem Solving						.321	.268	4.405*** (*P* <.001)	.178	.702
Social Support Seeking						.349	.264	4.342*** (*P* <.001)(*P* =.885)	-.191	.099
Avoidance						-.022	-.016	-.340*** (*P* =.734)	-.150	-.195
F	1.198					21.227^***^				
R^2^	.007					.235				
R^2^_Change_	.001					.224				

Note: ***P<.001; Dummy variables: Gender (male=1, female=0)
